# Serial dependence in time and numerosity perception is dimension-specific

**DOI:** 10.1167/jov.21.5.6

**Published:** 2021-05-06

**Authors:** Irene Togoli, Marta Fedele, Michele Fornaciai, Domenica Bueti

**Affiliations:** 1 International School for Advanced Studies (SISSA), Trieste, Italy; 2 International School for Advanced Studies (SISSA), Trieste, Italy; 3 KU Leuven, Faculty of Psychology and Educational Science, Leuven, Belgium; 4 International School for Advanced Studies (SISSA), Trieste, Italy; 5 International School for Advanced Studies (SISSA), Trieste, Italy

**Keywords:** serial dependence, numerosity perception, time perception, visual stability, magnitude integration

## Abstract

The perception of a visual event (e.g., a flock of birds) at the present moment can be biased by a previous perceptual experience (e.g., the perception of an earlier flock). Serial dependence is a perceptual bias whereby a current stimulus appears more similar to a previous one than it actually is. Whereas serial dependence emerges *within* several visual stimulus dimensions, whether it could simultaneously operate *across* different dimensions of the same stimulus (e.g., the numerosity and the duration of a visual pattern) remains unclear. Here we address this question by assessing the presence of serial dependence across duration and numerosity, two stimulus dimensions that are often associated and can bias each other. Participants performed either a duration or a numerosity discrimination task, in which they compared a constant reference with a variable test stimulus, varying along the task-relevant dimension (either duration or numerosity). Serial dependence was induced by a task-irrelevant inducer, that is, a stimulus presented before the reference and always varying in both duration and numerosity. The results show systematic serial dependencies only within the task-relevant stimulus dimension, that is, stimulus numerosity affects numerosity perception only, and duration affects duration perception only. Additionally, at least in the numerosity condition, the task-irrelevant dimension of the inducer (duration) had an opposite, repulsive effect. These findings thus show that attractive serial dependence operates in a highly specific fashion and does not transfer across different stimulus dimensions. Instead, the repulsive influence, possibly reflecting perceptual adaptation, can transfer from one dimension to another.

## Introduction

The stimuli we perceive are rarely isolated, but more often they are spatially surrounded and temporally preceded by others. The perception of a stimulus is indeed strongly dependent on both the spatial and the temporal context in which the stimulus is presented (e.g., see Schwartz, Hsu, & Dayan, 2007). In visual perception, serial dependence is a perceptual bias induced by the temporal context of the sensory experience. Specifically, it is an attractive bias in which the current stimulus appears mistakenly more similar to a previous one than it is in reality—much like an averaging of the features of successive stimuli. Serial dependence has been shown to be pervasive in vision, emerging in several visual domains spanning from relatively simple visual attributes such as orientation (Fischer & Whitney, 2014; Fritsche, Mostert, & de Lange, 2017; Pascucci, Mancuso, Santandrea, Della Libera, Plomp, & Chelazzi, 2019), position (Manassi, Liberman, Kosovicheva, Zhang, & Whitney, 2018), numerosity (Corbett, Fischer, & Whitney, 2011; Fornaciai & Park, 2018a; Fornaciai & Park, 2018b) and motion (Alais, Leung, & Van der Burg, 2017) to more complex features such as face identity (Liberman, Fischer, & Whitney, 2014), attractiveness (Xia, Leib, & Whitney, 2016), and visual variance (Suárez-Pinilla, Seth, & Roseboom, 2018).

However, despite these attractive biases having been documented across many studies and in several visual domains, the nature of serial dependence and the neural computations giving rise to it are still unclear. For instance, whether serial dependence arises from perceptual processing (e.g., Manassi et al., 2018), or it is determined by high-level, post-perceptual (i.e., decisional, cognitive) mechanisms (e.g., Fritsche et al., 2017; Bliss, Sun, & D'Esposito, 2017), is still unclear. Results from previous studies show, for instance, that serial dependence is highly dependent on attention and an active engagement with the task (Fischer & Whitney, 2014; Fornaciai & Park, 2018b; Fritsche & de Lange, 2019). For example, it has been shown that the effect even reverses (i.e., turning into a repulsive bias akin to adaptation) when the previous stimulus is not actively judged (Pascucci et al., 2019) or is made invisible by visual backward masking (Fornaciai & Park, 2019a). Finally, serial dependence seems also modulated by task instructions on a trial-by-trial basis: alternating different tasks over successive trials (for example, gender and attractiveness judgment of face stimuli; Van der Burg, Rhodes, & Alais, 2019) yields no serial dependence effect. The absence of serial dependence effects in this context suggests that the specific decision taken on a stimulus determines the information carried over to the next one. Overall, the current literature thus suggests that although potentially based on low-level perception, high-level processes and postperceptual mechanisms play an important role in generating serial dependence.

In line with the idea of high-level stimulus processing being involved in serial dependence, it has also been shown that serial dependence generalizes across stimuli with different low-level sensory properties (Fornaciai & Park, 2019b; see also Fischer, Czoschke, Peters, Rahm, Kaiser, & Bledowski, 2020). For example, in numerosity perception, the perceived numerosity of a dot array can be influenced by the number of preceding events in a sequence (i.e., by numerosity in a different presentation format). Serial dependence in this context seems to occur at a high-level processing stage, where numerical magnitude is represented in an abstract format (e.g., see for instance Arrighi, Togoli, & Burr, 2014). However, a question that remains so far unanswered is whether serial dependence could also generalize across different stimulus dimensions. Indeed, magnitude dimensions like space, time, and numerosity are thought to be processed by a high-level “generalized magnitude system,” which relies on a common neural code (or metric) to encode different information (e.g., Walsh, 2003; Bueti & Walsh, 2009). Cross-dimensional effects in a trial history context have been observed before. For instance, perceptual adaptation (see Kohn, 2007 for a review) has been shown to generalize across time and numerosity, although this effect is asymmetric (i.e., duration adaptation affects perceived numerosity, but in the opposite direction the effect is weaker or absent; Tsouli, Dumoulin, te Pas, & Van der Smagt, 2019; Tsouli et al., 2019). If serial dependence occurs at a similar abstract level of representation, it may then similarly generalize across different dimensions.

The similarity between attractive serial dependence and repulsive adaptation effects in vision is suggested by the observations that both phenomena emerge not only across “primary” visual dimensions such as orientation (Fischer & Whitney, 2014; He & MacLeod, 2001), numerosity (Fornaciai & Park, 2018b; Arrighi, Togoli, & Burr, 2014), position (Manassi et al., 2018; Whitaker, McGraw, & Levi, 1997), motion (Alais et al., 2017; Kohn & Movshon, 2003), or shape (Manassi Kristjánsson, & Whitney, 2019; Mattar, Olkkonen, Epstein, & Aguirre, 2018), but also across more complex features such as the summary statistics of a visual scene (Manassi, Liberman, Chaney, & Whitney, 2017; Corbett, Wurnitsch, Schwartz, & Whitney, 2012) and visual variance (Suárez-Pinilla et al., 2018; Maule & Franklin, 2020). Moreover, although adaptation and serial dependence may involve distinct physiological mechanisms, there is evidence that the same stimulus can induce either an attractive or a repulsive effect, depending on whether it was actively judged (Pascucci et al., 2019), or whether it was visible or suppressed by backward masking (Fornaciai & Park, 2019a; Fornaciai & Park, 2021). Considering these similarities between adaptation and serial dependence, one could ask: does serial dependence in magnitude perception occur at a level of analysis similar to adaptation, and thus generalize across different stimulus dimensions?

To address this question, here we focused on duration and numerosity. Duration and numerosity are indeed stimulus dimensions that are often naturally associated (i.e., the greater the number of people cueing at the supermarket till the longer the waiting time) and whose perception, when both dimensions are varied together, undergoes mutual biases (e.g., Arend, Cappelletti, & Henik, 2014; Javadi & Aichelburg, 2012; Xuan, Zhang, He, & Chen, 2007). For instance, in a duration discrimination task, the numerosity of a stimulus can bias its perceived duration, so that many items are perceived to last longer than fewer items, even if numerosity is completely task-irrelevant and not attended. These mutual biases between different dimensions support the idea of a common neural mechanism mediating the processing of magnitude information (e.g., Bueti & Walsh, 2009; Walsh, 2003). Duration and numerosity are also similarly sensitive to biases such as those provided by motion adaptation (Fornaciai, Arrighi, & Burr, 2016; Fornaciai, Togoli, & Arrighi, 2018), further supporting the idea that they share at least partially overlapping neural mechanisms and are encoded with similar metrics. In this context, our hypothesis is that serial dependence may operate at the level of a common representational system, predicting the existence of cross-dimensional effects.

Concerning the task, we used a numerosity and a duration discrimination task (in two separate sessions), in which we asked participants to discriminate either the numerosity or the duration of a constant reference stimulus from that of a variable test stimulus. Crucially, serial dependence was induced by a third *task-irrelevant* stimulus presented before the reference, which was always modulated in both duration and numerosity. This paradigm has been successfully used in multiple previous studies (e.g., Fornaciai & Park, 2018b; Fornaciai & Park, 2019a) and indeed has the advantage that no decision is required on the “past” stimulus (i.e., the inducer) for serial dependence to occur. This is very important in this context, because it allows us to assess the role of task context independently from the type of decision made on the past stimulus.

If serial dependence in magnitude perception operates at an abstract representational level, then we should expect an attractive bias across different dimensions irrespective of the task condition: an effect of both inducer numerosity and inducer duration on perceived numerosity in a numerosity task, and a similar effect of duration and numerosity in the duration discrimination task. If, on the other hand, serial dependence involves dimension-specific mechanisms, or it is specific for the task at hand, then we should expect only an effect of inducer duration on reference duration and of inducer numerosity on reference numerosity. Note that we predict a direct effect of the magnitude dimensions of the inducer on the magnitude perception of the reference – similar for instance to what has been observed in the case of adaptation (Tsouli, Dumoulin, te Pas, & van der Smagt, 2019; Tsouli, van der Smagt, Dumoulin, & te Pas, 2019)—and not an effect on the perception of the reference that is mediated by a biased perception of the inducer itself.

## Methods

### Participants

Twenty-eight participants took part in the study (20 females, mean age = 24.8, *SD* = 3.6). Participants were compensated for their time with 8 Euro/hour. All participants were naïve to the purpose of the experiment, had normal or corrected-to-normal vision, and signed the informed consent form before participating in the study. All the experimental procedures were approved by the SISSA ethical committee and were in line with the declaration of Helsinki. One participant was excluded after data analysis due to a too poor performance (Weber fraction, WF, > 1; see below *Data analysis*), leaving a total of 27 participants included in the results. The group size was determined a priori using a power analysis based on previous results (Fornaciai & Park, 2018b). As serial dependence in duration discrimination has not been tested before (but see Wehrman, Wearden, & Sowman, 2020, showing a decisional serial effect in a duration bisection task), we based the power analysis on the average effect size observed in numerosity perception across all the experiments included in Fornaciai and Park (2018b) (including only the conditions in which an effect was expected/observed). Based on these data, we estimated an average effect size (Cohen's *d*) of about 0.98. By assuming a power of 95% and a one-tailed distribution (based on the prediction of an attractive effect), the estimated group size was of 13 participants. Since the effect size of serial dependence in duration discrimination is not known, we conservatively doubled the estimated minimum group size, aiming to test at least 26 participants.

### Apparatus and stimuli

Stimuli were generated using the Psychophysics Toolbox (Brainard, 1997; Kleiner et al., 2007) for MatLab (version r2015b, The Mathworks, Inc.), and presented on a gamma-linearized LCD computer screen (running at 120 Hz) with a resolution of 1920 × 1080 pixels. All of the stimuli (with the exception of “catch” trials; see below) were arrays of black and white dots (50% black and 50% white for even numerosities; in case of odd numerosities the color of the exceeding dot was assigned randomly), presented on a gray background. In each trial, a sequence of three arrays of dots was presented: an “inducer” stimulus followed by a reference and a test stimulus. In all the experimental conditions, the inducer stimulus was similarly modulated in both numerosity and duration, according to two levels for each dimension (25 or 56 dots; 199 or 481 ms), for a total of four combinations. The reference stimulus was instead modulated according to the condition. In the numerosity task condition the reference had constant numerosity (37 dots) and was presented for a variable amount of time (199, 310, 481 ms). In the duration task condition, the reference stimulus had a constant duration (310 ms) and a variable numerosity (25, 37, 56 dots). Such a manipulation was introduced to assess the effect of magnitude integration (i.e., the biasing effect of different magnitudes on each other), and its interaction with serial dependence. The test stimulus was varied according to the specific condition as well. In the numerosity condition (where participants performed a numerosity discrimination task; see below *Procedure*), the test stimulus had variable numerosity (20, 25, 30, 37, 46, 56, or 69) and constant duration (310 ms). In the duration condition (where participants performed a duration discrimination task), the test stimulus had constant numerosity (37 dots) and variable duration (160, 199, 249, 310, 386, 481, or 600 ms). Overall, inducer, reference, and test stimuli were combined in a 4 × 3 × 7 design, with all the combinations presented an equal amount of times.

Besides the manipulations based on numerosity and duration, the dot array stimuli were also varied in several other nonnumerical attributes, that is, individual dot size, total area covered by the dots, field area, density. These nonnumerical attributes, captured by the dimensions (orthogonal to numerosity) of *size* and *spacing*, were modulated following the design used in previous studies (DeWind, Adams, Platt, & Brannon, 2015; Park, Dewind, Woldorff, & Brannon, 2016). More specifically, the dot array stimuli were constructed to span equal ranges in these three orthogonal dimensions (*numerosity*, *size*, and *spacing*). The size dimension was obtained by logarithmically scaling and combining the individual area of the dots and the total area covered by them. The spacing dimension was obtained by logarithmically scaling and combining the field area of the stimuli (i.e., the virtual circular area over which the dots are drawn) and their sparsity (i.e., the inverse of density). For more information about the stimulus construction procedure see DeWind et al., 2015; Fornaciai, Brannon, Woldorff, and Park, 2017; Park et al., 2016. The levels of nonnumerical dimensions were set as follows. The radius of the field area of the stimuli ranged from 200 to 320 pixels, corresponding to ∼4.3° to 8.6° of visual angle from a viewing distance of about 57 cm. The individual size of the dots (i.e., radius) ranged from 6 to 10 pixels, corresponding to ∼0.13° to 0.21° of visual angle. However, because the numerosity of the arrays and the duration of the stimuli were the only relevant features for the aim of the present study, the different levels of the nonnumerical dimensions were collapsed together for the data analysis. We nevertheless assessed the possible role of the different numerical and nonnumerical stimulus dimensions in the discrimination task. Namely, we assessed whether and to what extent the nonnumerical stimulus dimensions (i.e., size, spacing) of the inducer yielded serial dependence effects on the perception of the magnitudes of the reference. The results of this analysis did not show any significant effect (see Supplementary Figure S1).

### Procedure

Participants sat in a quiet and dimly lit room, with the screen placed at a distance of about 57 cm. Each participant performed two different conditions (i.e., duration and numerosity discrimination) of the experiment in two separated days, with the order of the conditions randomized across participants.

**Figure 1.**
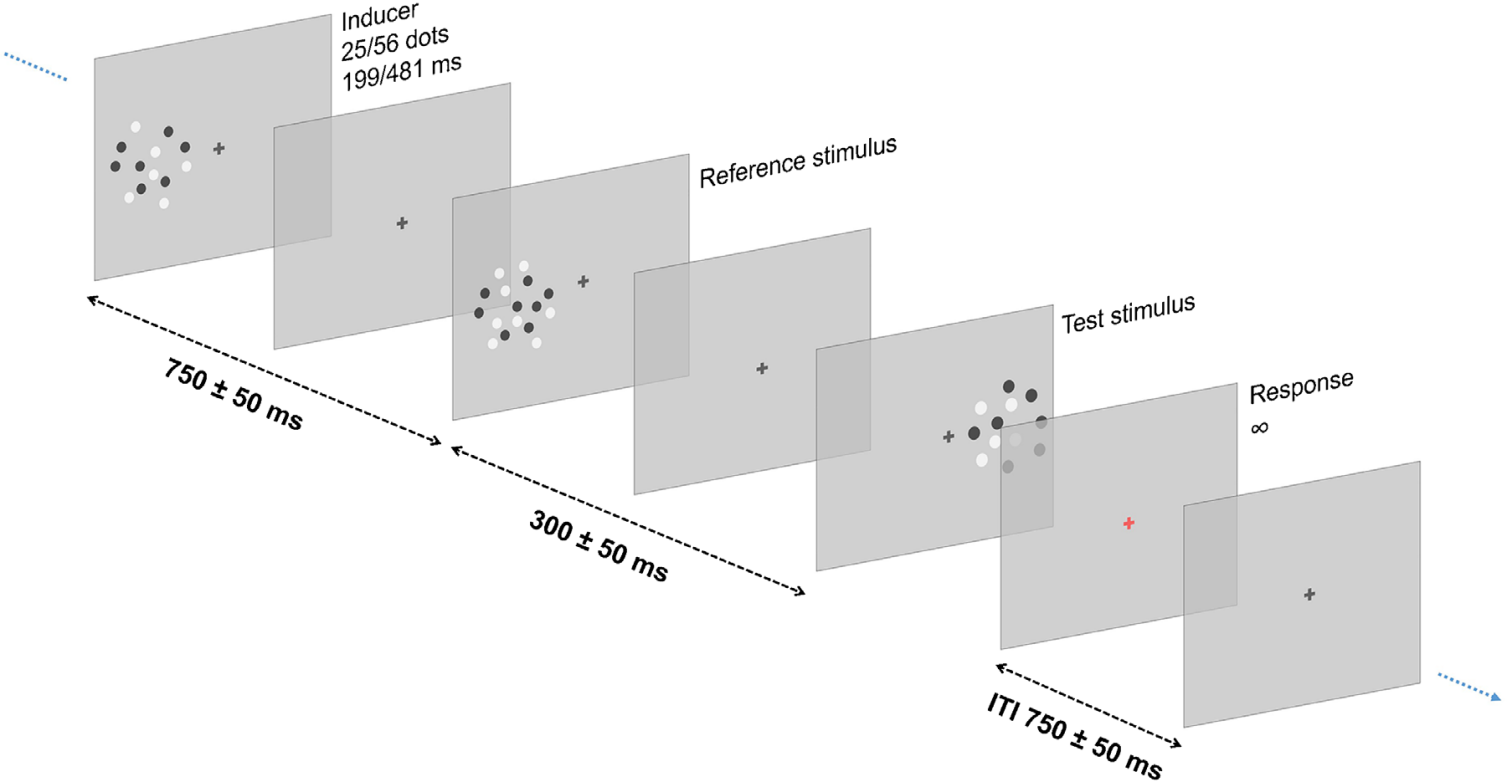
General experimental procedure. The experiment involved a numerosity and a duration task, performed in separate sessions. In both tasks, the sequence of stimuli presented in each trial included a first, task-irrelevant, inducer stimulus (with numerosity of either 25 or 56 dots, and duration of either 199 or 481 ms), followed by a reference (in the same position as the inducer) and test (in the opposite hemifield) stimulus. Participants had to compare reference and test and provide a response according to the task. In the numerosity task condition, the reference stimulus had constant numerosity (37 dots) and variable duration (199–481 ms), whereas the test had variable numerosity (20–69 dots) and constant duration (310 ms). In this condition, participants were asked to determine which stimulus between the reference and the test was more numerous. In the duration task condition, the reference had variable numerosity (25–56 dots) and constant duration (310 ms), whereas the test had constant numerosity (37 dots) and variable duration (160–600 ms). In this condition, participants were asked to determine whether the reference or the test lasted longer. In all cases, the inducer stimulus was irrelevant for the discrimination task. However, to encourage participants to pay attention to the inducer, a catch task was introduced in a small portion of trials (eight in each block; not shown in the figure). In a catch trial, the inducer stimulus was presented with blue and white dots (instead of black and white), and participants, instead of performing the discrimination task, had to press a different response key to signal the detection of the color change. Catch trials were excluded from the data analysis. Please note that for display purposes, stimuli are not depicted here in the actual dimension used in the experiment.

In the numerosity task condition, in each trial, participants had to compare the numerosity of a constant reference (37 dots) and a variable test (20–69 dots), presented sequentially (always in this order) in two different portions of the screen either on the left or on the right of a central fixation point (randomized across trials; center-to-center distance = 24° of visual angle). The reference was presented for a variable duration (199–481 ms), whereas the test was always presented for 310 ms. The interstimulus interval between the two stimuli was 300 ms with a variable jitter of ± 50 ms. A task irrelevant “inducer” stimulus was presented before the reference to induce serial dependence, with an inter-stimulus interval of 750 ms ± 50 ms. The position of the inducer was always the same as the reference stimulus (i.e., because it is supposed to affect the reference in a spatially localized fashion; Fornaciai & Park, 2018b), so either on the left or on the right portion of the screen. The inducer stimulus contained a variable number of dots (25 or 56 dots) and was presented for a variable duration (199 or 481 ms). After the presentation of the test, participants were instructed to provide a response as fast as they could by pressing the appropriate key on a standard keyboard. Participants had to decide whether the reference or the test stimulus contained more dots. After providing a response, the next trial started automatically after 750 ± 50 ms (see Figure 1 for a depiction of the experimental procedure).

In the duration task condition, the procedure was identical, except for the duration of the reference, which was kept constant (310 ms, whereas it was varied in numerosity between 25 and 56 dots), and for the test stimulus, which varied in duration (160–600 ms) and was kept constant in numerosity (37 dots). In this condition, participants were asked to decide whether the reference or the test stimulus lasted longer.

In both conditions, although the inducer stimulus was not relevant to perform the discrimination task, participants were asked to pay attention to all the stimuli appearing on the screen. To encourage the participants to pay attention also to the inducer stimulus (as it is required for serial dependence to emerge; see Fornaciai & Park, 2018b), a color oddball detection task was added to the experiment. In a small portion of trials (eight trials in each block) randomly interleaved within each block, the inducer stimulus was presented with blue (instead of black) and white dots. In those cases, participants were told to ignore the successive stimuli and press a button at the end of the trial to signal the detection of the catch stimulus. The color-oddball detection task was chosen to encourage participants to do not ignore the inducer, while avoiding to draw attention specifically to any of its magnitude dimensions (i.e., numerosity or duration). The detection rate in this task was on average (± *SD*) 97% ± 3.6%. In both conditions, participants never received any feedback about their responses.

Each condition of the experiment included 10 blocks of 92 trials, for a total of 10 repetitions of each combination of inducer, reference, and test. Note that in different parts of the analysis we collapsed together different dimensions of the stimuli to focus on the relevant ones, thus leading to a higher number of repetitions according to the specific analysis (see below *Data* *Analysis*). Each of the two conditions was completed in about 1.5 hours, with the two conditions performed in different days. Participants were free to take breaks during the experiment.

### Data analysis

The performance in the discrimination tasks was assessed separately for each task condition (i.e., duration and numerosity), obtaining measures of accuracy and precision at the single-subject level. Data analysis was performed by fitting a cumulative Gaussian function to all the data (i.e., proportion of “test more numerous” or “test longer” as a function of test magnitude) of each participant and each task condition. The average (± *SD*) goodness of fit (*R*^2^) of the fitting procedure was 0.62 ± 0.08 and 0.54 ± 0.14, respectively, for the numerosity and duration condition. The assessment of the serial dependence effect was based on the point of subjective equality (PSE), defined as the median of the cumulative Gaussian function fit to the data of each participant in a given condition, and reflecting the accuracy of the subjects. As a measure of precision, we first computed the just-noticeable difference (JND), defined as the difference in numerosity or duration between chance level responses and 75% correct responses. Moreover, the Weber fraction (WF = JND/PSE) was calculated as an additional measure of precision. WF measures were used to assess participants’ precision in the task and to exclude participants performing poorly in the discrimination tasks. As a threshold for exclusion, we used WF > 1, which led to the exclusion of one participant. Additionally, during the fitting procedure, a finger error (or lapse) rate correction (2%) was applied to reduce the noisiness of the data due to response errors or lapses of attention (Wichmann & Hill, 2001). This procedure involves a correction of the lower and the upper response probability bound. With a 2% correction, we thus have a response probability ranging from 0.02 to 0.98, instead of from 0 to 1. Repeated-measures analyses of variance (ANOVA) were carried out to compare the PSE in multiple conditions. Specifically, a two-way ANOVA with factors “inducer numerosity” and “inducer duration” was used to assess the serial dependence effect of the two dimensions of the inducers on the PSEs obtained in the numerosity and duration tasks (Figure 3). In the psychometric fitting procedure, PSEs were computed by collapsing together the different levels of the reference stimulus (i.e., the different reference durations in the numerosity task, and the reference numerosities in the duration task). This procedure allowed us to have a total of 30 repetitions for each combination of inducer and test stimuli. Additionally, to visualize the variability of the effect in the two conditions, we computed a serial dependence effect index as the difference in PSE between the two inducer magnitude levels within each dimension (i.e., PSE in the higher inducer magnitude condition minus the PSE obtained with a lower inducer magnitude; see Figure 4). To further compare the effect between the two task conditions, we normalized this serial dependence effect index and turned into percentage, according to the following formula:

(1) $$\begin{array}{ccc} & & \;\mathrm{Normalized}\;\mathrm{serial}\;\mathrm{dependence}\;\mathrm{index}\\ & & \;\;=\left(\left(\mathrm{PS}E_{\mathrm{HI}}-\mathrm{PS}E_{\mathrm{LOW}}\right)/\mathrm{PS}E_{\mathrm{LOW}}\right)\times 100;\; \end{array}$$

Where PSE_HI_ indicates the PSE obtained with the higher inducer magnitude (i.e., 56 dots or 481 ms, respectively for inducer numerosity and duration), and PSE_LOW_ indicates the PSE obtained with the lower magnitude inducer (25 dots or 199 ms).

A one-way ANOVA on individual PSE values was used to assess the effect of the reference magnitude manipulation (duration in the numerosity task and numerosity in the duration task; Figure 5). In this case, during the fitting procedure, we collapsed together the different levels of the inducer. This procedure leads to a total of 40 repetitions for each combination of reference and test stimuli. Finally, a three-way ANOVA was used to assess the interaction between serial dependence (i.e., inducer effect on the reference stimulus) and across magnitude manipulations (i.e., the effects of the different durations or numerosities of the reference stimulus on either the numerosity or the duration judgments respectively, Figure 6). In this case, the trials were divided according to both the inducer and the reference manipulation, for a total of 10 repetitions for each combination of inducer, reference, and test stimuli.

## Results

To address whether serial dependence generalizes across different magnitude dimensions, we used a numerosity and a duration discrimination task (performed in two separate sessions) of stimuli varying in both numerosity and duration. In the two tasks, participants had to decide which one of two stimuli, that is, a constant reference and a variable test, was more numerous (numerosity task) or lasted longer (duration task). In both cases, the reference and test stimuli were preceded by a task-irrelevant “inducer” stimulus to induce serial dependence. To assess serial dependence effects both within and across different stimulus dimensions, the inducer stimulus was modulated in both numerosity and duration. The effect of serial dependence was measured as the influence of the “inducer” on the perception of the subsequent reference stimulus, which was presented in the same spatial position. Specifically, we assessed how the perceived numerosity or duration of the reference stimulus, indexed by the point of subjective equality – PSE – varied as a function of the magnitude of the inducers.

**Figure 2.**
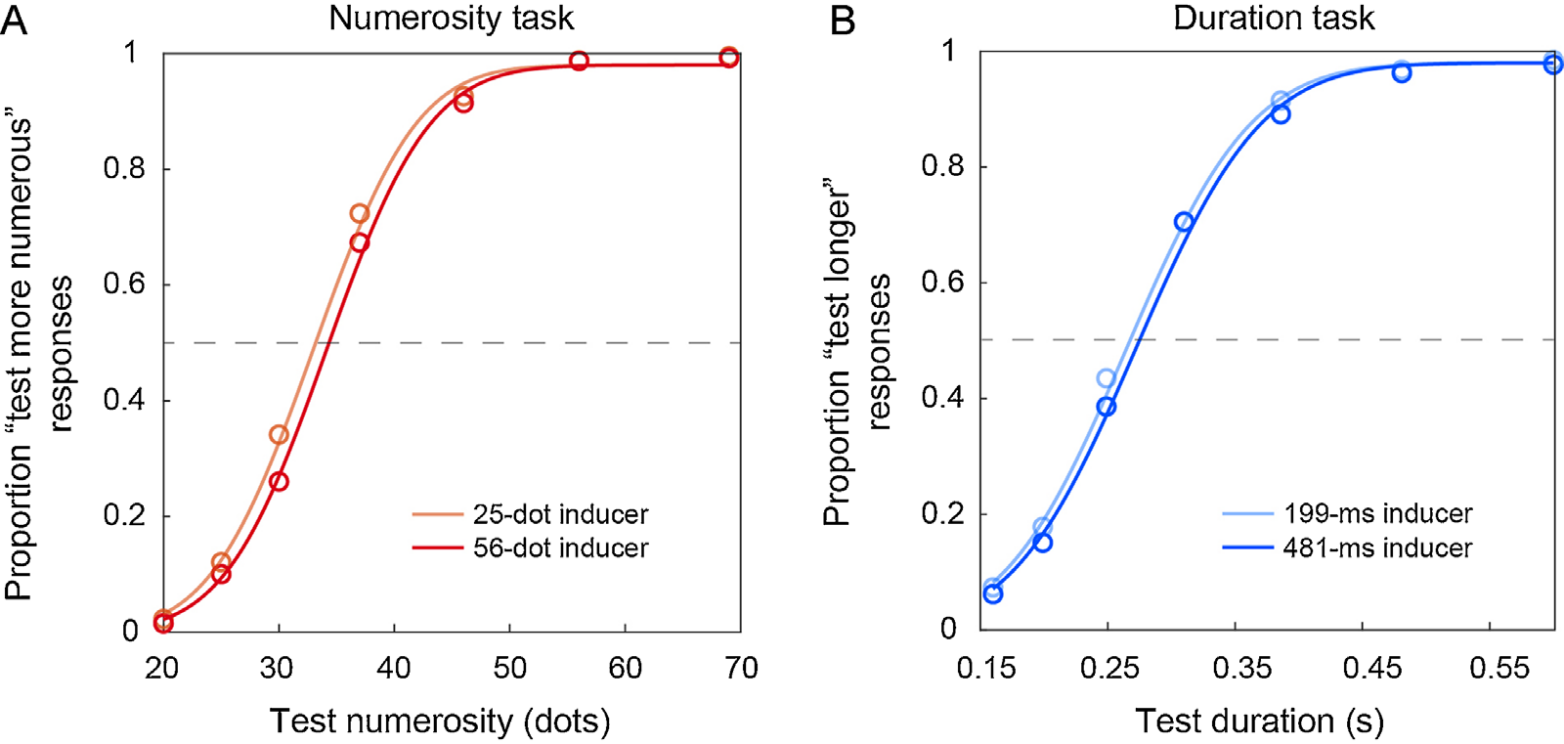
Average psychometric curves in the numerosity and duration task condition. (A) Average psychometric curves in the numerosity task condition, relative to the different levels of the inducer numerosity. (B) Average psychometric curves in the duration task condition, relative to the different levels of inducer duration. The horizontal dashed line indicates chance level responses.

Figure 2 shows the average psychometric curves in the two tasks, showing the serial dependence effects within the task-relevant dimension—that is, the effect of inducer numerosity in the numerosity task (Figure 2A), and the effect of inducer duration in the duration task (Figure 2B). As shown in the figure, in both conditions there is a small but clear difference in the curves as a function of the different inducer magnitudes. Namely, when the inducer magnitude (either numerosity or duration in the numerosity and duration task) was smaller than the reference, the relative curve appears shifted leftward compared to when the inducer magnitude was higher than the reference. This reflects a relative under- or overestimation of the reference magnitude according to the inducer, with an attractive pattern.

**Figure 3.**
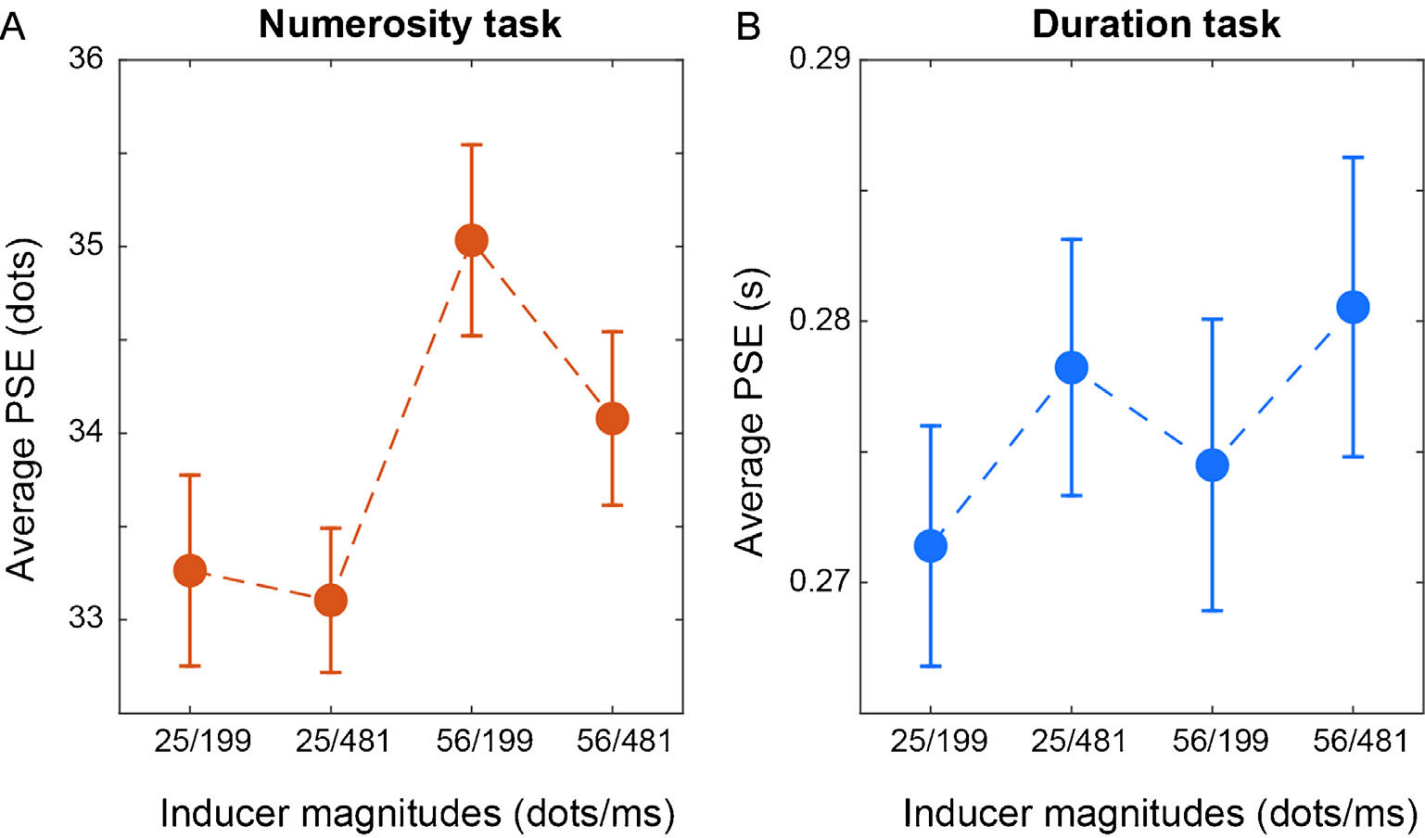
Serial dependence effects in the numerosity and duration task condition. (A) Average point of subjective equality (PSE) in the numerosity task condition as a function of the different combinations of inducer numerosity and duration. The results show an attractive effect of inducer numerosity (i.e., higher PSEs when the inducer had higher numerosity, compared to when the inducer contained fewer dots), and a smaller repulsive effect of inducer duration (i.e., the longer duration led to lower PSEs compared to the shorter duration). (B) Average PSE in the duration task condition as a function of the different combinations of inducer numerosity and duration. Here the results show an attractive effect of inducer duration, but no effect of inducer numerosity. Error bars are *SEM*.

This pattern of results was also statistically confirmed by a two-way repeated measures ANOVA with factors “inducer numerosity” and “inducer duration” performed on PSE measures (see the *Methods* section for details) obtained in both the numerosity and duration discrimination task. In the numerosity task, this analysis showed a significant main effect of inducer numerosity (*F*(1, 26) = 28.62, *p* < 0.001, η_p_^2^ = 0.27), a significant main effect of inducer duration (*F*(1, 26) = 4.72, *p* = 0.033, η_p_^2^ = 0.06), and no significant interaction between the two factors (*F*(1, 26) = 2.40, *p* = 0.125). As shown in Figure 3A, the inducer numerosity effect is clearly attractive, with a relative under- and overestimation for inducer numerosity respectively low or high (average effect in terms of difference in PSE between different inducer numerosities = ∼1.8 dots). The effect of inducer duration is instead repulsive—that is, the shorter inducer duration causes a slight increase in perceive numerosity, whereas the longer inducer duration results in a decrease in perceived numerosity (average effect = −0.56 dots). In the duration task (Figure 3B), on the other hand, we observed a main effect of inducer duration (*F*(1, 26) = 4.68, *p* = 0.034, η_p_^2^ = 0.06), but no main effect of the inducer numerosity (*F*(1, 26) = 0.83, *p* = 0.37) and no interaction between the two factors (*F*(1, 26) = 0.017, *p* = 0.89). In this case, the inducer duration had an attractive effect (average effect = ∼6.4 ms), whereas numerosity did not affect duration estimates.

Regarding the participants’ precision in the task, we assessed the pattern of Weber fractions (WFs) in each task and each inducer condition (data not shown). On average, Weber fractions were higher (i.e., poorer precision) in the duration compared to the numerosity task (0.16 ± 0.007 vs. 0.23 ± 0.024; paired *t*-test, *t*(26) = −2.94, *p* = 0.007). However, no difference in WF as a function of inducer numerosity or duration was observed in either task. In the numerosity task, a two-way repeated measures ANOVA (with factors “inducer numerosity” and “inducer duration”) on Weber fractions showed no main effect of either inducer numerosity or duration, and no interaction between the two factors (max *F* value = 3.39, min *p* value = 0.07). Similarly, no main effects and interactions were observed in the duration task (max *F* value = 1.28, min *p* value = 0.27).

**Figure 4.**
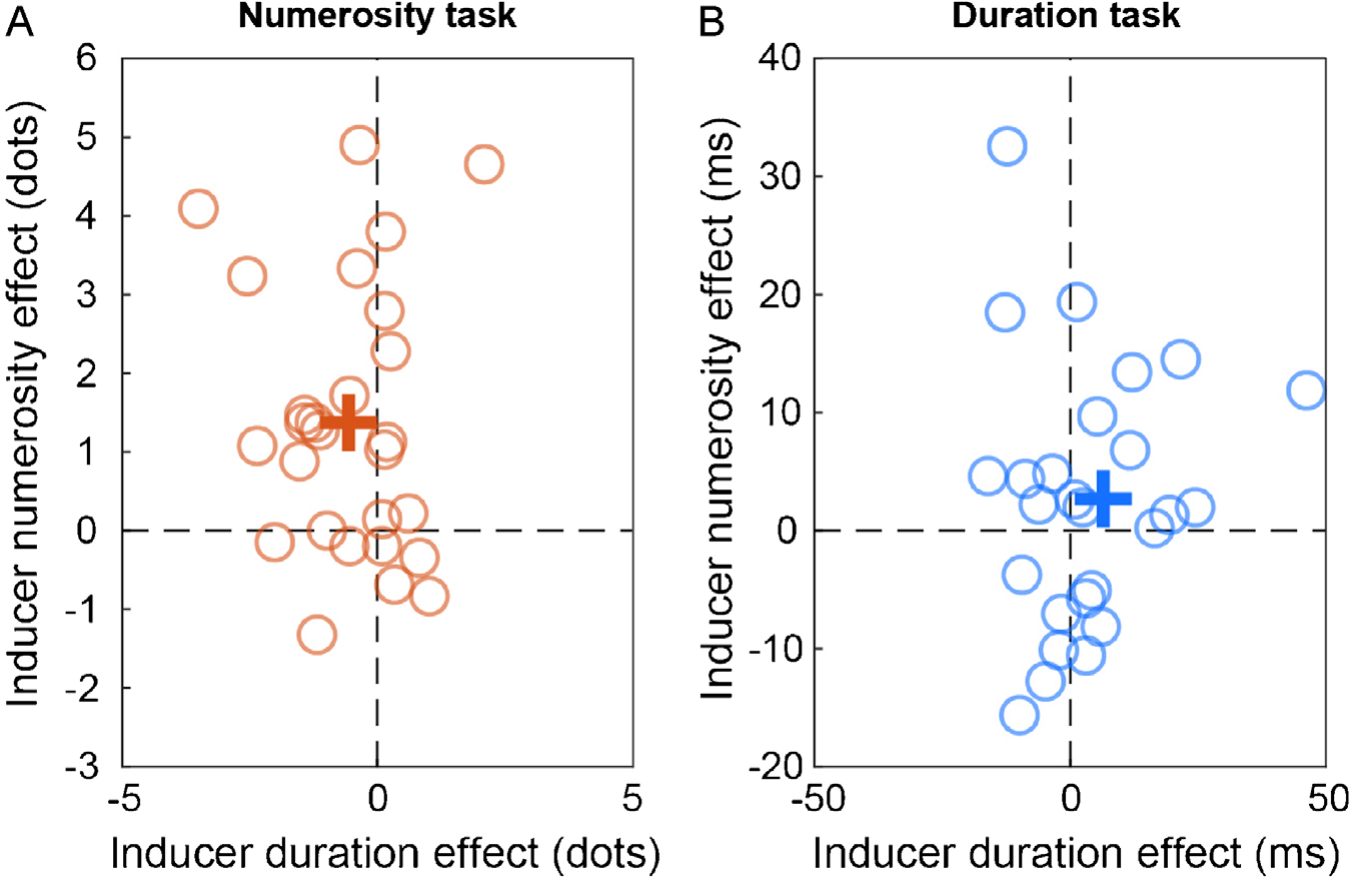
Effect of inducer numerosity and duration in the two task conditions. (A) Effect of inducer numerosity plotted against the effect of inducer duration, in the numerosity task condition. The effect here was calculated as the difference in point of subjective equality between either different inducer numerosities (i.e., high numerosity minus low numerosity, irrespective of duration) or different inducer durations (irrespective of numerosity). (B) Effect of inducer numerosity plotted against the effect of inducer duration in the duration task condition. Each empty symbol represents a different participant; bold crosses represent the average of all participants.

Figure 4 shows the serial dependence effects in the two tasks, for each individual subject. In this context, a measure of serial dependence effect was calculated as the difference between the different inducer numerosities (i.e., high numerosity minus low numerosity, irrespective of duration) or different inducer durations (i.e., long duration minus short duration, irrespective of numerosity). Regarding the effects in the numerosity task, although Figure 4A shows some variability across participants, the effect of inducer numerosity appears quite robust, with most of the data points laying in the upper part of the plot. Differently and in line with the previous analysis, the effect of inducer duration appears smaller, and the majority of the data points are shifted towards the negative (i.e., repulsive) axis. In the duration task (Figure 4B) we observed a larger variability compared to the numerosity task, with data points more distributed across positive and negative values, suggesting an overall less robust serial dependence effect. Overall, the effect of inducer duration in the duration task appears smaller in magnitude compared to the effect of the inducer numerosity in the numerosity task (normalized effect = 4.3% ± 5.5% vs. 2.8% ± 8.5%, respectively for numerosity and duration). This difference is however not statistically significant (*t*(26) = 0.82, *p* = 0.42).

**Figure 5.**
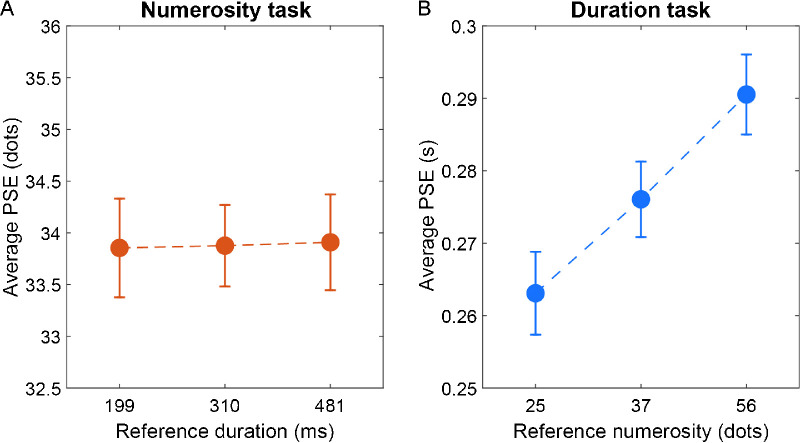
Effect of reference magnitude modulation. (A) Average point of subjective equality (PSE) in the numerosity discrimination task as a function of reference duration. The results showed no significant effect on the perceived numerosity of the reference as a function of duration. (B) Average PSE in the duration discrimination task as a function of the reference numerosity. In this condition, we observed a robust bias of the reference numerosity on perceived duration, with under- or overestimation according to the numerosity. Error bars are SEM.

Next, to assess the presence of perceptual biases across magnitude dimensions, we also checked whether and to what extent the task irrelevant magnitude changes of the reference stimulus influence the perception of the reference itself (Figure 5). The perceptual bias was again indexed by PSE. According to previous studies (Javadi & Aichelburg, 2012) we expect the task irrelevant changes in reference numerosity to bias the perceived duration in the duration task, and the task irrelevant changes in reference duration to bias the perceived numerosity in the numerosity task. Our predictions were partially confirmed by the results. In the numerosity task condition (Figure 5A), we did not observe any effect of reference duration on perceived numerosity (one-way repeated measure ANOVA with factor “reference duration”; *F*(1, 26) = 0.019, *p* = 0.98). Conversely, in the duration task condition (Figure 5B) we observed a robust influence of reference numerosity on perceived duration (one-way repeated measure ANOVA with factor “reference numerosity”; *F*(1, 26) = 12.73, *p* < 0.001, η_p_^2^ = 0.33).

**Figure 6.**
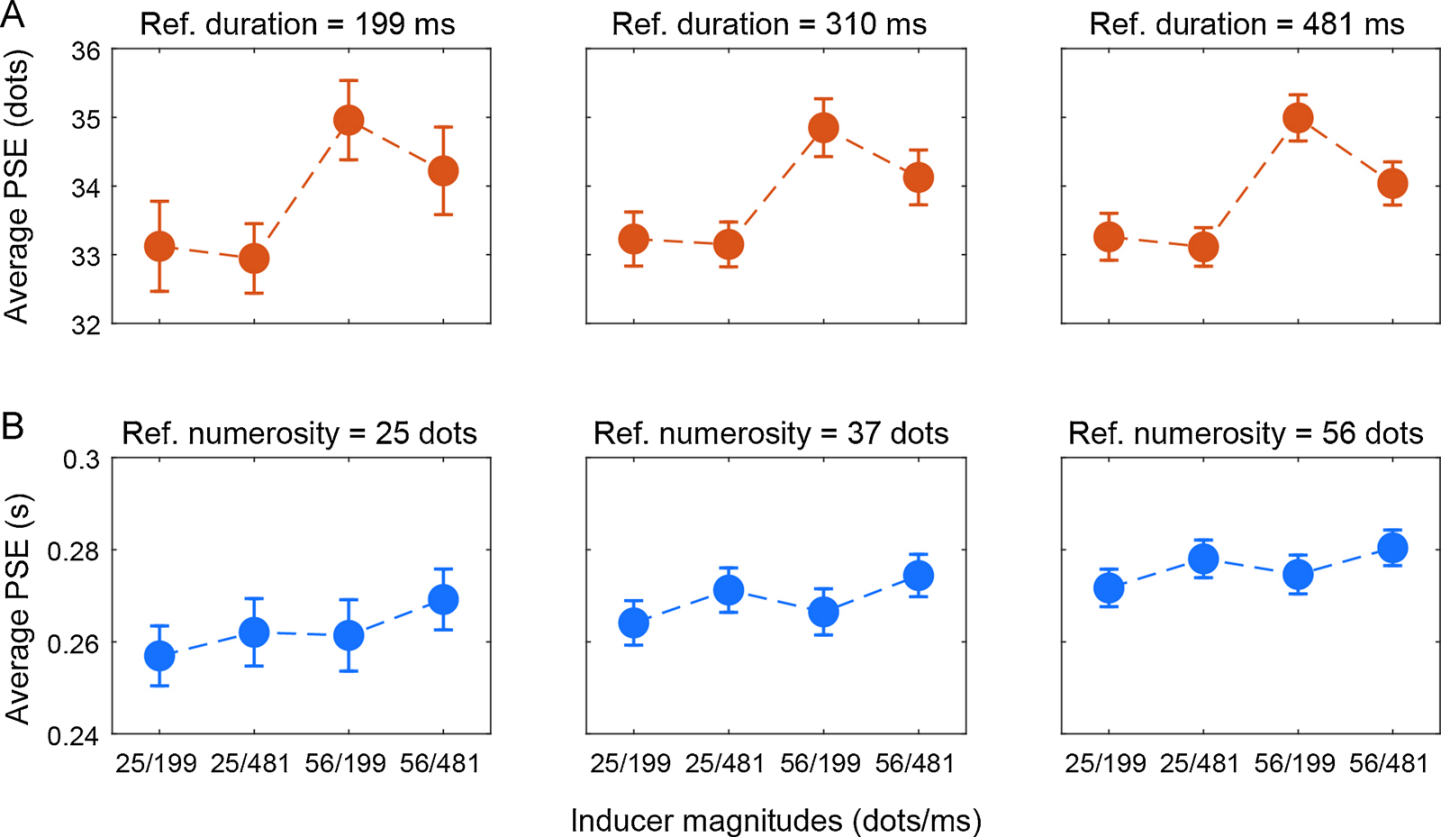
Serial dependence effect as a function of reference magnitude. (A) Average point of subjective equality (PSE) in the four inducer conditions in the numerosity discrimination task, divided according to three levels of reference duration. In this analysis, we again observed an attractive effect of the inducer numerosity on the perceived numerosity of the reference, and a small repulsive effect of the inducer duration. No significant effects were observed for the reference duration. (B) Average PSE in the four inducer conditions in the duration discrimination task, divided according to three levels of reference numerosity. Here we again observed an attractive effect of the inducer duration on the perceived duration of the reference, and no effect of the inducer numerosity. Additionally, we observed an effect of the reference numerosity, shifting the overall pattern of serial dependence effects in an additive fashion. Error bars are SEM.

Finally, we checked whether the serial dependence effects observed for perceived time and numerosity interacts with the perceptual bias induced by numerosity on time perception (Figure 6). Indeed, because magnitude integration affected the perceived magnitude of the reference stimulus (at least in the duration task), such distorted perception could have influenced serial dependence effects—that is, resulting in a stronger or weaker effect according to the perceived magnitude of the reference. To do so, for each condition (numerosity and duration) we divided the data according to both the different combinations of inducer magnitude and the different levels of reference stimulus. To characterize a possible interaction between the two biases, we used a three-way repeated measure ANOVA with factor “inducer numerosity,” “inducer duration,” and “reference magnitude” (either duration in the numerosity condition or numerosity in the duration condition). In the numerosity task condition (Figure 6A), again we observed a main effect of inducer numerosity (*F*(1, 26) = 34.04, *p* < 0.001, η_p_^2^ = 0.11) and of inducer duration (*F*(1, 26) = 5.86, *p* = 0.016, η_p_^2^ = 0.02), but no effect of the reference duration (*F*(1, 26) = 0.03, *p* = 0.97) and no interactions (neither two-way nor three-way) between any of the factors (all *F* values < 3.16, *p* values > 0.08). In the duration task condition (Figure 6B), we found a main effect of inducer duration (*F*(1, 26) = 3.94, *p* = 0.048, η_p_^2^ = 0.01), no effect of inducer numerosity (*F*(1, 26) = 0.76, *p* = 0.38), and a significant main effect of the reference numerosity (*F*(1, 26) = 28.26, *p* < 0.001, η_p_^2^ = 0.16). Again, we did not observe any interaction between any of the factors (all *F* values < 0.33, *p* values > 0.72). Overall, these results are in line with the previous analyses, showing the effects of inducer numerosity (attractive) and inducer duration (repulsive) in the numerosity task, and the effect of inducer duration in the duration task. Additionally, this analysis shows that when the perception of the reference is also biased by manipulating another task-irrelevant dimension (i.e., numerosity in the duration task), this bias adds to serial dependence without interacting with it.

## Discussion

In the present work, we asked whether serial dependence generalizes across different stimulus dimensions, or whether it is a dimension-specific effect. To address this question, we leveraged on the link between numerosity and duration, testing whether the magnitude of a previous, task-irrelevant, stimulus (either numerosity or duration) could bias the perceived magnitude of a subsequent one (i.e., numerosity in a numerosity task and duration in a duration task). Importantly, while previous studies (Fritsche & de Lange, 2019; Van der Burg et al., 2019) show that the decision made on a past stimulus determines which information is carried over to the next stimulus (i.e., if you previously judged the gender of a face, there is no bias on the perceived attractiveness of a subsequent stimulus; Van der Burg et al., 2019), here we measured this effect using a task-irrelevant inducer stimulus to rule out the effect of past decisions in determining serial dependence.

Contrary to the hypothesis of mutual, cross-dimensional serial effects between magnitude dimensions, we did not observe serial dependence across duration and numerosity. What we observed instead was the presence of attractive serial dependence only within the stimulus dimension that was currently relevant for the task. Namely, when participants were asked to judge numerosity, we observed attractive effects of the inducer numerosity only, and when they were asked to judge duration, an attractive effect of the inducer duration only. However, our results did not show only dimension-specific effects. Indeed, although we did not observe cross-dimensional attractive serial dependence effects, we observed a cross-dimensional repulsive bias of the inducer duration on the perceived numerosity of the reference in the numerosity discrimination task. This is particularly important, as it supports the idea that cross-dimensional biases are, in principle, measurable with the present paradigm. What is, however, the nature of this effect? The repulsive nature of this bias suggests a perceptual adaptation effect (Kohn, 2007). Previous results also support this idea. Indeed, it has been shown than duration adaptation biases the perceived numerosity of a subsequent stimulus in a repulsive fashion (Tsouli, Dumoulin, et al., 2019; Tsouli, van der Smagt, et al., 2019). Additionally, repulsive adaptation effects across numerosity and duration also appear to be asymmetric, with an opposite pattern compared to magnitude integration, that is, duration adaptation affects perceived numerosity, but numerosity adaptation does not affect perceived time (Tsouli, Dumoulin, et al., 2019). This is again in line with the present results and supports the idea that the observed repulsive bias might in fact be a perceptual adaptation effect.

Considering the present results, the central question thus is why serial dependence is limited to a single stimulus dimension, whereas adaptation can transfer across dimensions (at least from duration to numerosity). We thought about two possible accounts of this result. The first possibility is that serial dependence occurs, in this context, at a more limited level of abstraction compared to adaptation. Intuitively, this might seem surprising, because adaptation is usually considered a low-level physiological process (e.g., Kohn, 2007), whereas serial dependence very often shows the hallmarks of a much higher-level effect (e.g., Fornaciai & Park, 2019b; Pascucci et al., 2019). However, adaptation effects in magnitude perception often show high-level properties. For instance, duration adaptation is not selective for the spatial position of the stimuli, highlighting a neural substrate beyond early, topographically-organized, visual areas (Maarseveen, Hogendoorn, Verstraten, & Paffen, 2017). Numerosity adaptation generalizes not only across different stimulus formats, but also across different sensory modalities (Arrighi, Togoli, & Burr, 2014) and across the perceptual and the motor systems (Anobile, Arrighi, Togoli, & Burr, 2016; Anobile, Domenici, Togoli, Burr, & Arrighi, 2020; Togoli, Crollen, Arrighi, & Collignon, 2020). On the other hand, the effect of serial dependence in numerosity perception appears to be much more limited compared to adaptation, because it does not transfer across different sensory modalities (i.e., vision and audition; Fornaciai & Park, 2019b). In line with these last empirical observations, the absence of cross-dimensional attractive biases in our study might be explained by assuming that serial dependence occurs within a dimension-specific processing pathway, upstream to the putative generalized magnitude system. The second possibility instead concerns the role of task set in limiting serial dependence to the task-relevant dimension only. Indeed, in our paradigm we probed duration and numerosity in two separate tasks, where each of them was the only dimension relevant for the task. The role of task-set in serial dependence has been shown by previous studies. For instance, Van der Burg and colleagues (2019), in the context of face perception, showed that when participants were required to judge different aspects of a face in successive trials (i.e., gender in one trial and attractiveness in the next) serial dependence effects disappear. In a different study, Fritsche and de Lange (2019) showed that feature-based attention driven by a task strongly affects the magnitude of the serial dependence effect. Namely, when participants judged the size, instead of the orientation, of a grating stimulus, the serial dependence effect on the orientation of the next stimulus was reduced. Differently from these previous studies, however, our task did not require an active judgement of the “past” stimulus inducing serial dependence (i.e., the inducer). Our results would thus further suggest that an explicit decision is not necessarily needed to influence the pattern of serial dependence, but the “task set” itself would be sufficient to modulate the effect. However, this only concerns the attractive effect between two successive stimuli, and not the repulsive effects such as adaptation (which may even be facilitated by the suppression of serial dependence; see also Fornaciai & Park, 2019a).

Besides the influence of the type of task performed by participants, also attention to a specific magnitude dimension of the inducer can play a role in the present results. Indeed, even in the absence of an explicit task concerning the inducer, participants may have implicitly paid attention to the inducer magnitude dimension relevant for the subsequent task. In fact, the concept of “task set” includes the process of paying attention to the stimuli or dimensions relevant for the task at hand (Rogers & Monsell, 1995). If attention played a role, our results might suggest that attention to a particular stimulus dimension modulates the pattern of serial dependence even implicitly, without an active task performed on the “past” stimulus (i.e., the inducer). Overall, our results cannot however distinguish whether the absence of cross-dimensional attractive effects is determined by the mechanisms of serial dependence being implemented at a dimension-specific processing stage, or because of an inhibitory role of the task set. Indeed, our paradigm could not conclusively pinpoint whether cross-dimensional serial dependence is not in fact possible due to its specific nature, or whether it could be possible in principle, but it is actively limited to the task-relevant stimulus dimensions. A crucial task for future studies would thus be to disentangle the role of task-relevance and attention from the specificity of the serial dependence effect per se.

Is the absence of a cross-dimensional serial dependence effect observed here (absence of evidence) truly an evidence of serial dependence being a dimension-specific effect (evidence of absence)? Indeed, one may argue that the effect may still be there, but too small to be measured, or even absent due to the asymmetric pattern of magnitude integration effect. If the effect was based for instance on a biased representation of the inducer due to magnitude integration (i.e., inducer numerosity biasing its perceived duration, and consequently providing an effect based on the distorted duration), the effect would likely be too small to be measured. However, it is important to consider in this context that magnitude integration and serial dependence may be mediated by different and potentially dissociable processes. Indeed, whereas magnitude integration concerns the representation of the different dimensions of the same stimulus, serial dependence relates to the effect of the stimulus history on the current sensory perception. Although serial dependence effects modulated by a biased representation of the past stimulus are possible (Fornaciai & Park, 2021), our current task, which does not require any magnitude judgment of the inducer, has not been designed to fully capture this interaction. However, as shown in Figure 3, the lack of a congruency effect across the different combinations of inducer magnitudes (i.e., increased effect when the inducer has either lowest duration and numerosity, or highest duration and numerosity) in the duration task, and the opposite pattern observed in the numerosity task (i.e., leading to the repulsive effect of inducer duration on reference numerosity), seems against a magnitude integration effect at the inducer level. Additionally, as mentioned above, our paradigm could successfully capture cross-dimensional effects, although only in the opposite, repulsive, direction. This further supports the idea that our results provide a genuine evidence for the absence of cross-dimensional attractive serial dependence.

Regarding the asymmetric magnitude integration effect observed between duration and numerosity at the level of the reference stimulus, although in contrast with a few previous studies (e.g., Javadi & Aichelburg, 2012), it is, however, not unusual. Indeed, the interaction between different magnitudes is often found to be asymmetrical, with time usually described as the most vulnerable dimension (e.g., Dormal & Pesenti, 2013; Arend et al., 2014). In a recent study from our group (Togoli, Fornaciai, & Bueti, 2020), we have linked asymmetries in magnitude integration to the processing dynamics of visual information. Information like numerosity is processed in a very fast fashion (e.g. Fornaciai & Park, 2018c), and virtually completed within ∼250 ms from stimulus onset. Duration information, instead, could be represented only after the entire interval has elapsed. In the presence of a relatively long interval like the one used here (310 ms), numerosity could easily interfere with duration processing during the interval, but duration would be represented too late to retroactively affect the numerosity representation. In this study (Togoli, Fornaciai, & Bueti, 2020) we have tested this hypothesis and we have shown that if one reduces the temporal lag between the numerosity and time representation by making numerosity to unfold over time (asking participants to judge the average numerosity of a series of dots arrays), the integration becomes symmetrical.

Another important question in this context is the following: would the dimension-specificity observed here generalize to serial dependence effects in different dimensions and tasks? Indeed, there are quite marked differences in the properties of serial dependence effects reported in different studies. For instance, serial dependence in orientation reproduction is tightly tuned to stimulus similarity (Fischer & Whitney, 2014), has a broad spatial selectivity (Collins, 2019; Fischer & Whitney, 2014), depends on past decisions (Pascucci et al., 2019), and it is sensitive to contextual stimulus information (Fischer, Czoschke, Peters, Rahm, Kaiser, & Bledowski, 2020). The serial dependence effect in numerosity perception, on the other hand, seems not particularly sensitive to stimulus similarity (Fornaciai & Park, 2020a), shows a much tighter spatial selectivity (Fornaciai & Park, 2018b), and works in the absence of decisions and even when two successive stimuli are completely different (Fornaciai & Park, 2018b, 2019b). Such differences in the effect across different stimulus dimensions and tasks raise the possibility that serial dependence may be supported by multiple independent mechanisms rather than a common mechanism operating according to the same computational principles in different contexts. This in turn suggests that the dimension-specificity shown in the present study may be limited to the discrimination paradigms used here, or perhaps dependent on the relation between the stimulus dimensions that we tested (i.e., numerosity and duration). In fact, the hypothesis of a cross-dimensional effect is driven by the intrinsic link between numerosity and duration, while no effect would be expected a priori for not-closely-related dimensions, like for instance orientation and color or size. Testing the generalizability of the present effects to different magnitude dimensions and tasks represents an interesting possibility for future studies. Additionally, if the task set played a role in determining the observed effects, the fact that participants performed the two tasks in separate sessions might have influenced the selectivity of the serial dependence effect observed here. With different tasks intermixed within the same session, the serial dependence effect could more easily transfer from one dimension to another. However, the opposite could also be possible: having different interleaved tasks might even limit the effect within each dimension, similarly to what has been observed by Van der Burg et al. (2019). Testing the effect of interleaved tasks thus represents another interesting possibility that should be addressed in future studies.

How do these results fit with the current models of serial dependence? The finding of dimension-specific attractive effects is for instance in line with the idea of a “continuity field” involved in mediating visual stability, as proposed by Fischer and Whitney (2014). In this context, thus, the integration of information within the continuity field would either occur at a processing level upstream to the generalized magnitude system, or could be modulated by the current task set. The present results are however in contrast with the idea that serial dependence mediates the stability of the whole visual scene (Manassi, Liberman, Chaney, & Whitney, 2017) especially in the case of complex environments. Our results are also in line with the alternative, but not mutually exclusive idea that serial dependence originates at post-perceptual levels but propagates to early visual areas via feedback signals from higher to lower level visual regions (Fornaciai & Park, 2018a, 2019b, 2019c). In general, our results are consistent with the idea of serial dependence as a signature of visual stability (e.g., Fischer & Whitney, 2014) and further suggest that the integration of past and present information occurs in a highly selective fashion.

To conclude, our results provide new evidence for a very specific role of serial dependence in magnitude perception. Here we showed that, even in a paradigm not requiring an active judgment on a past stimulus, the attractive bias typical of serial dependence does not generalize across different stimulus dimensions, as for instance adaptation (at least in some cases) does. Moreover, we also show a repulsive bias induced by the task-irrelevant visual feature, supporting the idea that repulsive adaptation effects more easily transfer across dimensions. Overall, our results provide evidence for a dissociation between attractive serial dependence and repulsive adaptation effects and show that serial dependence operates in a highly selective fashion.

## Supplementary Material

Supplement 1
