# Supplementary material for: Serial dependence in time and numerosity perception is dimension-specific
Source: J Vis. 2021 May 6;21(5):6. doi: 10.1167/jov.21.5.6 (PMC8107483; doi:10.1167/jov.21.5.6)
Supplement: Supplement 1 [file jovi-21-5-6_s001.pdf]

SUPPLEMENTARY MATERIALS

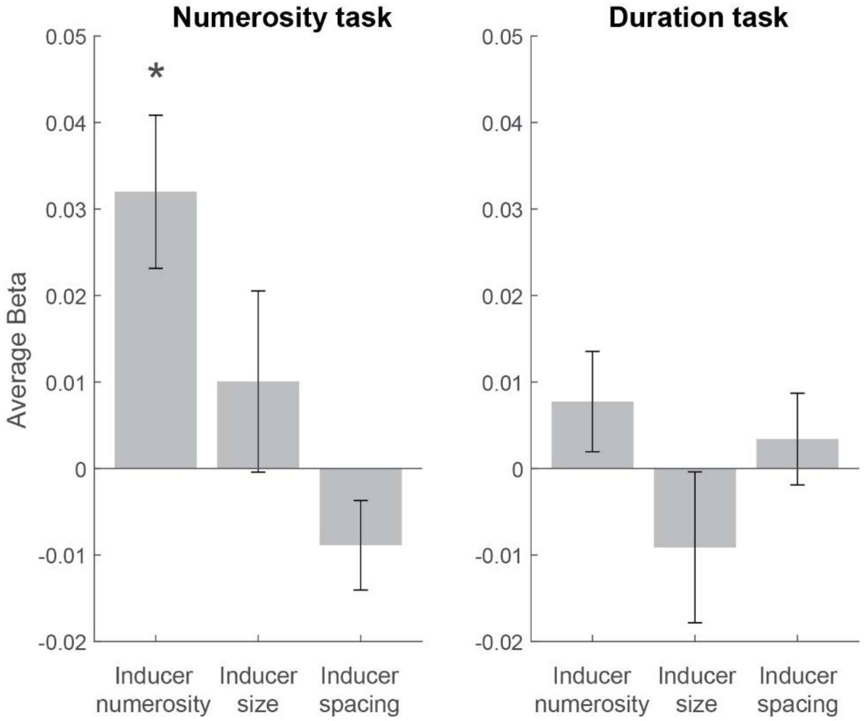

Figure S1. Serial effects induced by numerical and non-numerical stimulus attributes. *Results of the non-linear regression analysis assessing the effect of inducer numerosity, size, and spacing on performance in the numerosity (left panel) and duration (right panel) task condition. The results are reported in terms of average beta value, indexing the extent to which any of the magnitudes examined contributed to the judgment in the discrimination task. The significance of these effects was tested with a one-sample t-test against the null hypothesis of no effect (i.e., average beta = 0). Error bars are SEM. * $p < 0.05$.*

Although numerosity and duration represented the main stimulus dimensions examined in the present study, other non-numerical stimulus dimensions were concurrently modulated, following the same procedure used by DeWind et al. (2015) and Park et al. (2016). We thus further assessed whether the modulation of non-numerical dimensions at the level of the inducer yielded serial effects on the reference perceived magnitude.

To address this possibility, we directly tested for an effect of the different numerical and non-numerical magnitude dimensions of the inducer in the numerosity and duration discrimination task. Namely, we performed a non-linear regression analysis assessing the trial-by-trial effect of the different inducer magnitudes on the behavioural response in the discrimination task. We thus entered the log numerosity of the inducer, and the log of its dimensions of "size" and "spacing" computed as in DeWind et al. (2015) and Park et al. (2016). In this context, positive beta values resulting from the regression analysis indicate an attractive effect – i.e., the probability of responding that the reference has a bigger magnitude than the test increases as the relative magnitude of the inducer increases – while negative beta values would index an opposite, repulsive effect. The results of this regression analysis are shown in Fig. S1. In the numerosity

task condition, the results only show a significant attractive effect of numerosity (one-sample t-test, $t(26) = 3.62$, $p = 0.0013$, Cohen's $d = 0.70$), while no significant effect of size and spacing was observed ($t(26) = 0.96$, $p = 0.35$ and $t(26) = -1.71$, $p = 0.09$, respectively). In the duration task condition, no effect of either numerosity ($t(26) = 1.33$, $p = 0.19$), size ($t(26) = -1.04$, $p = 0.31$), or spacing ($t(26) = 0.64$, $p = 0.53$) was observed.

These results suggest that the different non-numerical magnitude dimensions of the inducer stimulus did not yield any significance serial dependence effect, and did not substantially influence the judgment in the discrimination tasks.

REFERENCES

DeWind, N. K., Adams, G. K., Platt, M. L., & Brannon, E. M. (2015). Modeling the approximate number system to quantify the contribution of visual stimulus features. Cognition, 142, 247–265. https://doi.org/10.1016/j.cognition.2015.05.016

Park, J., Dewind, N. K., Woldorff, M. G., & Brannon, E. M. (2016). Rapid and Direct Encoding of Numerosity in the Visual Stream. Cerebral Cortex, 26(2), 748–763. https://doi.org/10.1093/cercor/bhv017
